# Metabolomics analysis of the potential mechanism of Yi-Guan-Jian decoction to reverse bone loss in glucocorticoid-induced osteoporosis

**DOI:** 10.1186/s13018-023-03778-6

**Published:** 2023-06-05

**Authors:** Mengxing Yin, Dezhi Zhou, Fu Jia, Xiaosan Su, Xiufang Li, Ruifen Sun, Junmin Li

**Affiliations:** 1 Yunnan University of Chinese Medicine, Kunming, Yunnan, China; 2 Department of Orthopedics, Yan’an Hospital Affiliated to Kunming Medical University, Kunming, Yunnan, China; 3 West Yunnan University of Applied Sciences, Dali, Yunnan, China

**Keywords:** Bone metabolism, Glucocorticoid-induced osteoporosis, Metabolomics, Taurine and hypotaurine metabolism, Yi-Guan-Jian decoction

## Abstract

**Background:**

Glucocorticoid-induced osteoporosis (GIOP) is a disease in which long-term use of glucocorticoid causes bone loss, deterioration of bone microstructure and fracture. Currently, clinical drugs targeting this disease have certain side effects. There is still a need to find effective drugs with fewer side effects. The theory of traditional Chinese medicine suggests that YGJ has therapeutic effect on GIOP, but it has not been explained. Therefore, this study aims to explore the protective effect of YGJ on GIOP mouse models and elucidate the underlying mechanism through LC–MS-based metabolomics analysis.

**Methods:**

The general condition of 8 week age male C57BL/6J mice was recorded after 8 weeks of treatment with dexamethasone (DEX) and YGJ. Bone-related parameters and bone morphology were determined by Micro-CT. HE staining was used to observe the pathological changes of bone tissue. Serum levels of bone metabolism markers were detected by ELISA. Liver metabolomics analysis was conducted to search for the significant markers of anti-GIOP of YGJ and the metabolic pathway affecting it.

**Results:**

After treatment, YGJ significantly reversed the weight loss caused by DEX; increase the number of bone trabecular in ROI region, significantly improve the bone-related parameters of GIOP mice, and increase the levels of alkaline phosphatase and osteocalcin. In the study of metabolic mechanism, YGJ reversed 24 potential markers in GIOP mice. These included cortisol, 3-hydroxybutyric acid, taurine, esculin and uric acid, which are closely associated with osteoporosis. Topological analysis results showed that YGJ had the most significant effect on taurine and hypotaurine metabolism, with − log10 (*P*) > 2.0 and Impact > 0.4.

**Conclusions:**

Yi-Guan-Jian decoction can increase bone density and improve bone microstructure by regulating the levels of alkaline phosphatase and osteocalcin and reverse bone loss in GIOP mouse model. The underlying metabolic mechanism may be related to taurine and hypotaurine metabolic pathway.

**Supplementary Information:**

The online version contains supplementary material available at 10.1186/s13018-023-03778-6.

## Background

Glucocorticoids are widely used clinically in patients suffering from a variety of chronic diseases, such as rheumatoid arthritis, inflammatory bowel disease, allergic diseases and organ transplantation and chronic obstructive pulmonary disease [1, 2]. Osteoporosis is a common and serious complication of glucocorticoid therapy, characterized by reduced bone mass, decreased osteoblast activity and deterioration of microstructure [3], which eventually leads to fracture or osteonecrosis if left untreated. There are current pharmacological treatments for GIOP, with calcium and vitamin D supplements, bisphosphonates (alendronate, risedronate and zoledronate), therapeutic monoclonal antibodies (denosumab), N-terminal active fragments of parathyroid hormone (teriparatide), and selective estrogen receptor modulators (raloxifene, lasofoxifene and bazedoxifene). An analysis of common osteoporosis agents suggests that denosumab, pamidronate and zoledronate all showed a favorable increase in spine-related bone density after treatment, while denosumab has a better effect with t respect to the hip and femur [4].But studies have found that these medications can come with a range of side effects, even bone loss and fractures may occur rapidly after discontinuation of the drug [5]; therefore, the need to continue the search for new drugs that are safe and effective with few side effects for the treatment of GIOP is of great clinical significance.

In the knowledge system of Chinese traditional medicine (TCM), GIOP is in accordance with the characteristics of TCM that "medicine causes disease". Exogenous glucocorticoids are pungent and warm medicines, and long-term use will deplete qi and harm yin, resulting in deficiency of kidney essence and inability to nourish liver yin and blood, so in the treatment of GIOP, TCM considers it very important and effective to nourish liver and kidney [6]. Yi-Guan-Jian decoction (YGJ)comes from Xu Mingyi Lei'an, is mainly for the treatment of liver and kidney yin deficiency, which fits well with the TCM etiology of GIOP. However, no studies have confirmed the feasibility of YGJ for GIOP treatment. In order to explore the role of YGJ in disease development, a systematic analysis using GIOP mouse models is required.

Metabolomics, the comprehensive analysis of small molecule metabolites in cells, tissues or whole organisms [7], which are integral to cellular function and involved in multiple enzyme-catalyzed reactions. While upstream bioperturbation can cause a series of metabolomic changes, it is rich in biological information and is considered to be the most predictive phenotype [8]. Metabolomics has become a hot topic in osteoporosis research, and the use of metabolomic techniques for early detection, diagnosis, and treatment of patients with OP has been beneficial in reducing the prevalence and improving the quality of life of patients.

In this study, GIOP models were used to characterize the therapeutic effect of YGJ on GIOP, and micro-CT, HE staining and ELISA were performed to determine the indicators of bone formation and bone resorption in serum for pharmacodynamic studies. In order to explore the potential active ingredients, LC–MS was used to identify the metabolic components and find the metabolic pathways through which they function. The study aims to lay the groundwork for the clinical use of YGJ in the treatment of GIOP, where it is expected to be a potentially effective drug to some extent.

## Methods

### Animals, grouping and model establishment

**Fig. 1:**
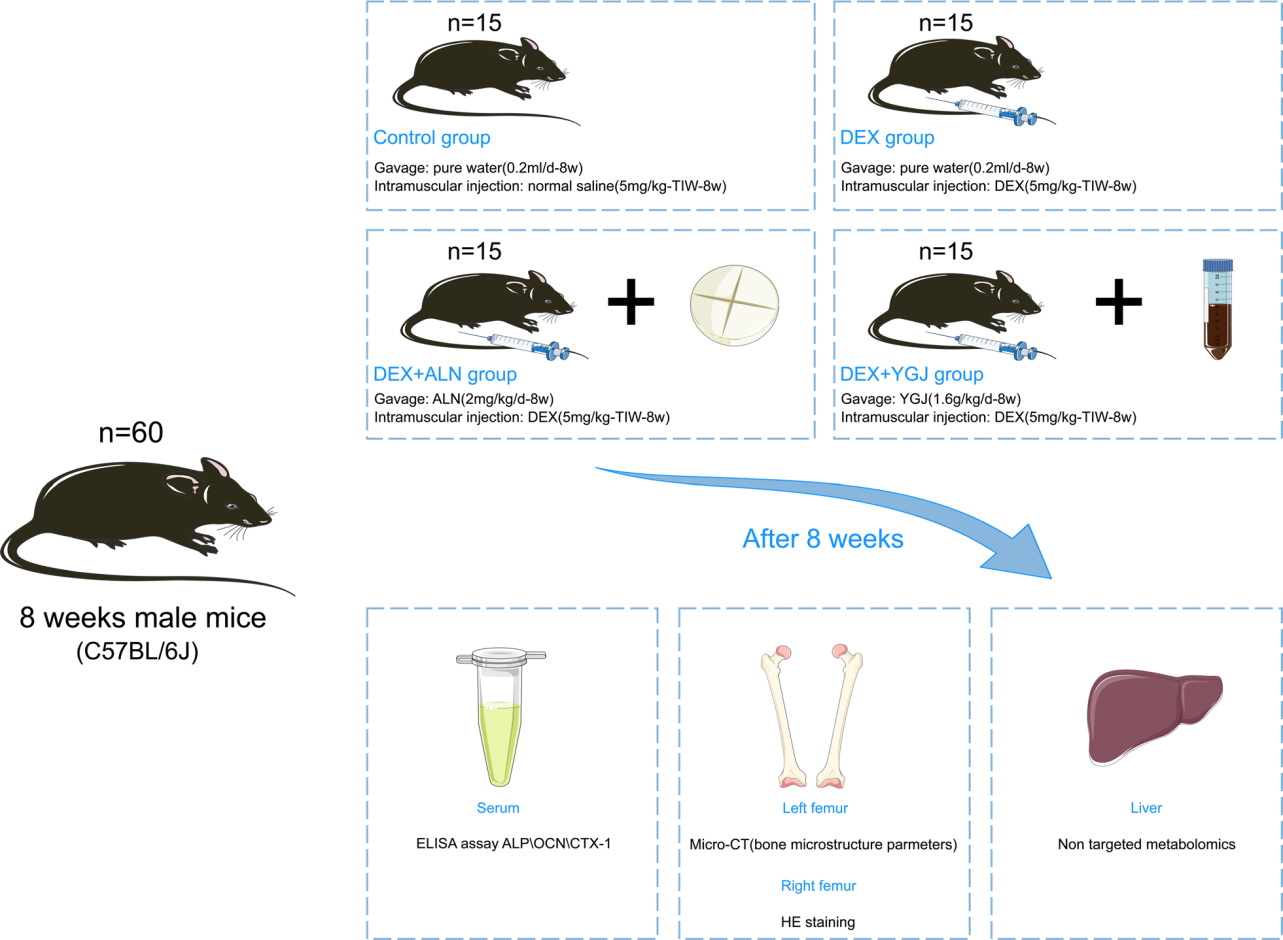
Schematic diagram of animal grouping and intervention

Sixty male C57BL/6J mice (age, 8 weeks; weight, 21 ~ 23 g) were acclimatized and fed for one week, during which they were kept at a constant temperature of about 25℃, with normal light and dark cycles, and provided with normal drinking water and normal animal chow (purchased from Hunan, China, Chushang Technology). They were randomly divided into four groups: Control, DEX, DEX + ALN and DEX + YGJ group. Mice in the Control group were interested in gavage with pure water once a day (0.2 mL each time) for 8 weeks. The other three groups were given the GIOP model, and DEX (5 mg/kg) was alternately injected into the bilateral quadriceps muscle three times a week for 8 weeks. At the same time, a gavage intervention was performed. The DEX group was given gavage with pure water every day (0.2 mL each time). In the DEX + ALN group, alendronate sodium tablets were dissolved in pure water and administered interactively at a daily dose of 2 mg/kg. In the DEX + YGJ group, granules were dissolved in pure water at a dose of 1.6 g/kg daily for gavage treatment (schematic diagram of animal grouping and intervention is shown in Fig. 1).

### Drugs and reagents

YGJ (Kunming Traditional Chinese Medicine Hospital, Kunming, China); dexamethasone sodium phosphate injection (lot no. 22204071; Suicheng Pharmaceutical Co., Henan, China); alendronate sodium tablets (lot no. U003019; MSD, Savio Industrial S.r.L, Italy); 4% paraformaldehyde tissue fixative (Biosharp, Anhui, China); use of Purified Water System (Kunming, China) to obtain purified water; alkaline phosphatase (ALP) ELISA kit, Osteocalcin (OCN) ELISA kit, C-terminal telopeptide of type-I collagen (CTX-I) ELISA kit (Shanghai Preferred Biotechnology Co., Ltd., Shanghai, China).

### General observations and sample collection

Weighing was performed every Monday during weeks 1–8. On the last day of the 8th week, fasting and water deprivation were performed. The blood was obtained from the eyes early the next morning. The liver was stripped and stored at − 80 °C. Both femurs were taken, and saline-soaked gauze was wrapped around the right femur and stored directly at − 80 °C. The left femur was rinsed and soaked in 4% paraformaldehyde and stored at normal temperature.

### Micro-CT detection of bone-related parameters

Scanning of the left femur of mice was done on a Venus Micro-CT (VNC-102) device from PINGSENG Healthcare Inc. The device uses conical beam CT, an imaging technique capable of high resolution. At the beginning of the scan, the mouse femur is placed in the sample chamber, the scanning tube voltage is set to 60 kV, the tube current is set to 180 uA, the detector and the sphere tube are rotated 360° around the central axis of the sample chamber during the scan, and 10,000 projections are made over the scan area, the entire scan time is 2000s. After the image is captured by the detector, it is transmitted to the computer, and then the image is reverse-reconstructed by FDK method on Avatar software (version 1.7.2, PINGSENG Healthcare Inc.) with the pixel size of 6um × 6um × 8um. The bone trabecular analysis area was chosen to be a 0.5-mm-long area 1 mm below the growth plate, with bone parameters such as BMD, BMC, Tb. Sp, Tb. N, SMI, Conn.D, BV/TV, and BS/TV which were measured and analyzed.

### Observation of the pathology of the femoral section

The right femur was removed from the refrigerator and placed in 4% paraformaldehyde for 72 h for fixation. The bone was decalcified with 20% EDTA (pH 7.4) for 4 weeks at room temperature and then embedded in paraffin, and the paraffin sections were cut into 5-μm-thick sections, stained with hematoxylin and eosin according to HE staining method, observed and photographed under a slide scanner, and analyzed for average positive optical density values using ImageJ software (version 2.0).

### Measurement of biochemical markers in serum

Blood samples were taken and allowed to stand for 2 h. After centrifugation at 3000 rpm for 10 min at 4 °C, upper serum samples were obtained. For use, they were removed from the − 80 °C refrigerator to thaw on ice, centrifuged and serum bone formation markers (ALP and OCN) and bone resorption markers (CTX-I) concentrations were measured according to the assay kits listed in the Materials section.

### Non-targeted metabolomics analysis

A 100-mg mouse liver sample ground in liquid nitrogen was placed in an EP tube, and 500 μL of 80% aqueous methanol was added; the sample was eddied and shaken, left in an ice bath for 5 min and centrifuged at 15,000 g for 20 min at 4℃. A certain amount of the supernatant was diluted with mass spectrometry grade water to 53% methanol; the supernatant was collected at 15,000 g for 20 min at 4℃ and centrifuged using Vanquish UHPLC (Thermo Fisher, Germany) chromatograph with Hypesil Gold column (100 × 2.1 mm, 1.9 μm; Thermo Fisher, USA) for liquid chromatography detection. Column temperature: 40 °C, flow rate: 0.2 mL/min, positive mode: mobile phase A: 0.1% formic acid, mobile phase B: methanol, negative mode: mobile phase A: 5 mM ammonium acetate, pH 9.0, mobile phase B: methanol.

The Q Exactive™ HF (Thermo Fisher, Germany) mass spectrometer was used, and the scan range was selected from m/z 100–1500. The ESI source was set as follows: spray voltage: 3.5 kV; sheath gas flow rate: 35 psi; aux gas flow rate: 10L/min; capillary temp: 320 °C; S-lens RF level: 60; aux gas heater temp: 350 °C; polarity: positive, negative; MS/MS secondary scan is a data-dependent scan.

### Statistical analysis and processing of metabolic data

Data conforming to normal distribution were expressed as mean ± SD, and statistical analysis was performed using SPSS 16.0 software. For data conforming to normal distribution and homogeneity, one-way analysis of variance (ANOVA) was used to compare the overall mean differences between multiple groups of data, and different data were subjected to the least significant difference (LSD) difference test for significance of differences between two groups. For data that do not conform to a normal distribution, the Kruskal–Wallis test was used to compare the statistical significance of the groups. GraphPad Prism v9.0 (La Jolla, CA, USA) was used for data visualization analysis. *P* < 0.05 indicates that the difference is statistically significant.

The data (.raw) files were imported into CD3.1 library search software for processing, and simple screening of parameters such as retention time and mass-to-charge ratio was performed for each metabolite, setting retention time deviation of 0.2 min and mass deviation of 5 ppm for peak alignment of different samples to make identification more accurate, followed by setting mass deviation of 5 ppm, signal intensity deviation of 30%, signal-to-noise ratio of 3, minimum signal intensity, additive and ion information for peak extraction, as well as quantification of peak area, then integration of target ions, followed by molecular ion peak and fragment ion for molecular formula prediction and comparison with mzCloud, mzVault and Masslist databases, and normalization of raw quantitative results. Finally, the identification and relative quantification results of metabolites were obtained. The data processing was partially performed on the Linux operating system (CentOS version 6.6) and software R and Python. The identified metabolites were annotated using the KEGG database (https://www.genome.jp/kegg/pathway.html), HMDB database (https://hmdb.ca/metabolites), and MetaboAnalyst (http://www.metaboanalyst.ca/) and KEGG pathway database (http://www.genome.jp/kegg/) for pathway analysis of potential biomarkers.

## Results

### Effect of YGJ on bone-related parameters in GIOP mice

**Fig. 2:**
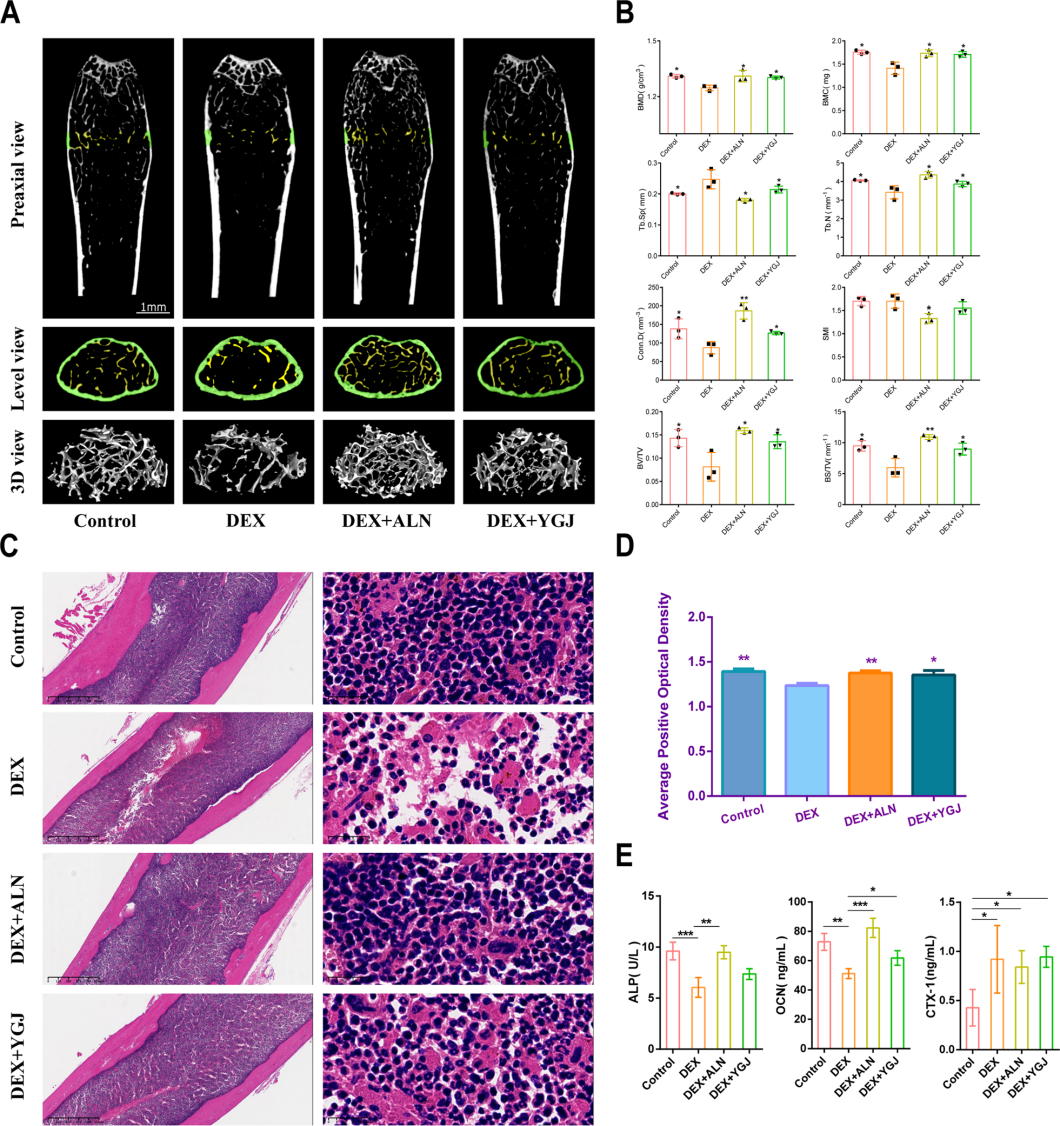
Analysis of bone morphology and serum biochemical markers in GIOP mice. **A** Micro-CT preaxial, level, and 3D scan views; **B** quantitative study of Micro-CT bone parameters, including BMD: bone density, BMC: bone mineral content, Tb.Sp: bone trabecular separation (the average width of medullary cavity between bone trabecular), Tb.N: bone trabecular number, SMI: structural pattern index (describing the ratio of lamellar and rod-shaped structure in trabecular structure, If the structure is mainly lamellar structure, SMI is close to 0; otherwise, it is a rod-shaped trabecular structure. It was close to 3), Conn.D: bone trabecular connection density (indicating the number of connections between bone trabecular reticular structures per cubic millimeter volume), BV/TV: bone volume fraction (ratio of bone tissue volume to tissue volume in the selected area), BS/TV: bone surface area tissue volume ratio (bone surface area density); **C** HE-stained section of the right femur of mice; **D** average positive optical density value; **E** effects of YGJ on ALP, OCN and CTX-I levels in GIOP mice.*P < 0.05, **P < 0.01, ***P < 0.001

As shown in Fig. 2A, the number of bone trabecular fractures was significantly reduced in the DEX group compared to the Control, DEX + ALN, and DEX + YGJ groups, and there were larger cavities in the bone tissue. After treatment with YGJ and ALN, the microstructure of the femur was observed to improve in GIOP mice, with increased bone trabeculae in the ROI region. In addition to this, the DEX group showed significant differences in BMD, BMC, Tb. Sp, Tb. N, Conn.D, BV/TV, BS/TV, increased trabecular gap, and decreased bone junction density compared with the Control group. The BMD, BMC, Tb. N, Conn.D, BV/TV and BS/TV were improved, and the trabecular space was reduced and bone loss was reversed after drug treatment (Fig. 2B).

### Effect of YGJ on histopathology of the femur bone of GIOP mice

In order to explore the pathological changes of YGJ on the histomorphology of GIOP mice after 8 weeks, the number, structure and average positive optical density values of bone trabecular were observed, respectively. The results are shown in Fig. 2C, where the control group revealed dense structural tissue with intact trabecular morphology, homogeneous structure and normal marrow cavities. The DEX group exhibits a blurred and fractured trabecular morphology, increased interstitials, and significantly enlarged marrow cavities. The trabecular morphology of GIOP mice treated with YGJ and ALN improved to varying degrees, with their bone cells aligned evenly and neatly and the marrow cavity reduced. To further confirm the effect of drug administration on the reduction of bone loss using imaging analysis software, the results are demonstrated in Fig. 2D. The control, DEX + ALN and DEX + YGJ groups have significantly higher mean positive optical density values than the DEX group.

### Effect of YGJ on bone formation and bone resorption in GIOP mice

As shown in Fig. 2E, the serum ALP results of mice in the DEX group showed significantly lower ALP levels and impaired bone formation. The GIOP mice treated with ALN for 8 weeks showed a clear rebound in ALP levels. The GIOP mice had higher levels of ALP after 8 weeks of YGJ than the DEX group, and although there was no significant difference. It also ameliorated bone hypoplasia to some extent. In another measure of bone formation, the remaining three groups improved the reduction of OCN level to varying degrees compared to the DEX group. In the bone resorption serum index, CTX-I occurred a trend of enhanced bone resorption function in the remaining three groups after DEX injection compared with the Control group, and this function was not improved after administration.

### Metabolomics analysis of mouse liver

**Fig. 3:**
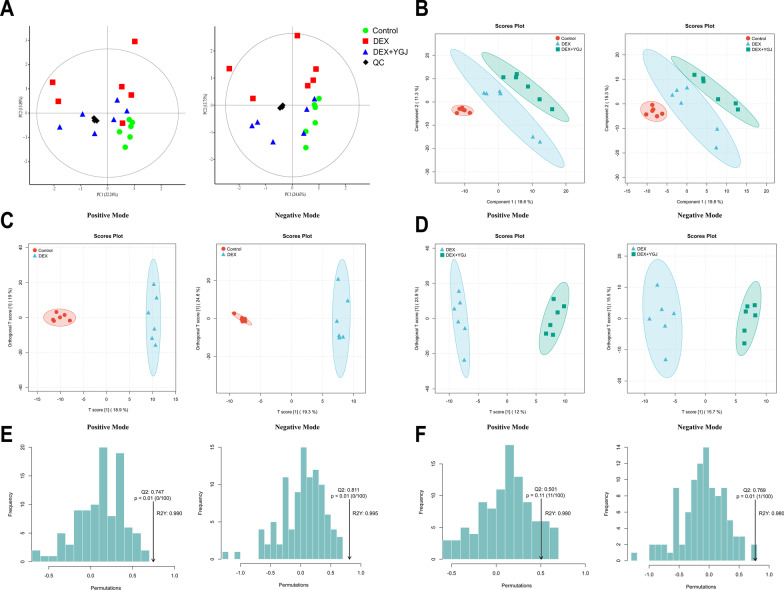
Multivariate statistical analysis of the liver of mice based on LC–MS metabolomics. **A** PCA score plots in positive and negative ion modes; **B** PLS-DA score plots in positive and negative ion mode; **C** OPLS-DA scores of DEX group and Control group in positive and negative ion mode; **D** OPLS-DA scores of DEX group and DEX + YGJ group in positive and negative ion mode; **E** OPLS-DA substitution test plots of DEX group and Control group in positive and negative ion mode; **F** OPLS-DA substitution test plots of DEX group and DEX + YGJ group in positive and negative ion mode

Six separate liver tissue samples from each group were fully scanned using LC–MS in both positive and negative ionic modes under optimal conditions. The QC samples are mixed with equal amounts of all samples and inserted at the beginning, end and some intermediate locations during signal data acquisition to record signal drift. Figure 3A shows that the QC sample is clustered in both positive and negative ionic modes, demonstrating the good effect of the correction. The three subgroups were more obviously distributed in different regions in the PCA score plot, and the DEX + YGJ group was closer to the Control group than the DEX group in terms of position distance. That indicating the metabolite composition structure of the two subgroups was similar and had more obvious differences from the GIOP mouse model. The PLS-DA and OPLS-DA models were used to investigate the effects of metabolite patterns on the liver after DEX and YGJ interventions. As shown in Fig. 3B, the PLS-DA model discriminated better in both ionization modes, there is a clear separation trend in the metabolic profiles of the liver in the three groups. The results showing the metabolites are disordered in the mice after DEX injection. The OPLS-DA further confirmed that the difference between the DEX and Control group and the DEX + YGJ was clear. In each mode, R^2^Y > 0.9, Q^2^ > 0.5, the value of which was close to 1, indicating that the DEX group was different from the other two groups in terms of metabolic profile without overfitting, and the differentiation effect was credible (Fig. 3C–F).

### Screening for significantly different metabolites

**Fig. 4:**
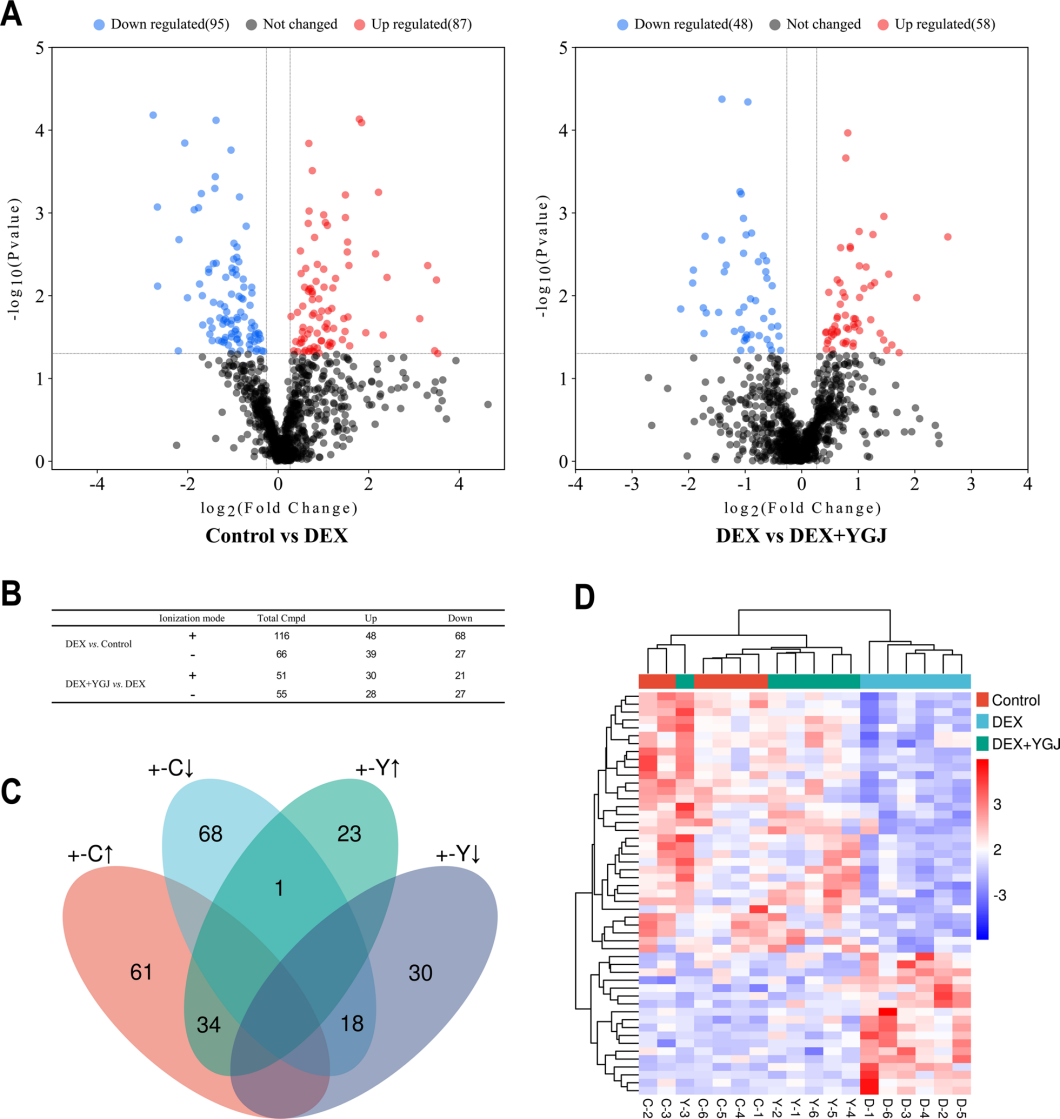
Screening to identify significant markers of YGJ reversal of metabolism in GIOP mice. **A** Volcano plot analysis of DEX group versus Control and DEX + YGJ groups in positive and negative ion mode; **B** summary of metabolites of significant differences between DEX group and Control group and DEX + YGJ group in positive and negative ion mode (+ : positive ion mode, − : negative ion mode); **C** Venn diagram of significant markers of YGJ balance in positive and negative ion mode (↑: up-regulation, ↓: down-regulation); **D** cluster heat map analysis of 52 significant markers among the three groups

A total of 606 metabolites were detected in the positive ion mode, and 364 metabolites were detected in the negative ion mode by detection and calculation. The screening of significantly different metabolites was based on the two conditions of Variable Importance for Projection (VIP) value greater than 1.0 in the OPLS-DA analysis model, combined with t-test *P* < 0.05. Based on the above steps, 182 metabolites were detected between the Control and DEX groups, and 106 different metabolites were detected between the DEX + YGJ and the DEX groups. The volcano plots were plotted according to the condition of *P* < 0.05 and FC ≥ 1.2 (Fig. 4A). In the positive and negative ion mode, 87 metabolites were up-regulated and 95 metabolites were down-regulated after DEX injection, and 58 metabolites were up-regulated and 48 metabolites were down-regulated after the metabolic profile changed after YGJ intervention (Fig. 4B). In order to find significant markers of GIOP treated with YGJ, metabolites with the same changes in the DEX + YGJ and Control groups under the two modalities were overlapped for analysis, and the results are shown in Fig. 4C. A total of 52 metabolites were back-regulated by YGJ. The 52 significant markers derived above were imported into the heat map clustering analysis (Fig. 4D), among which 34 metabolites were up-regulated and 18 were down-regulated.

**Fig. 5:**
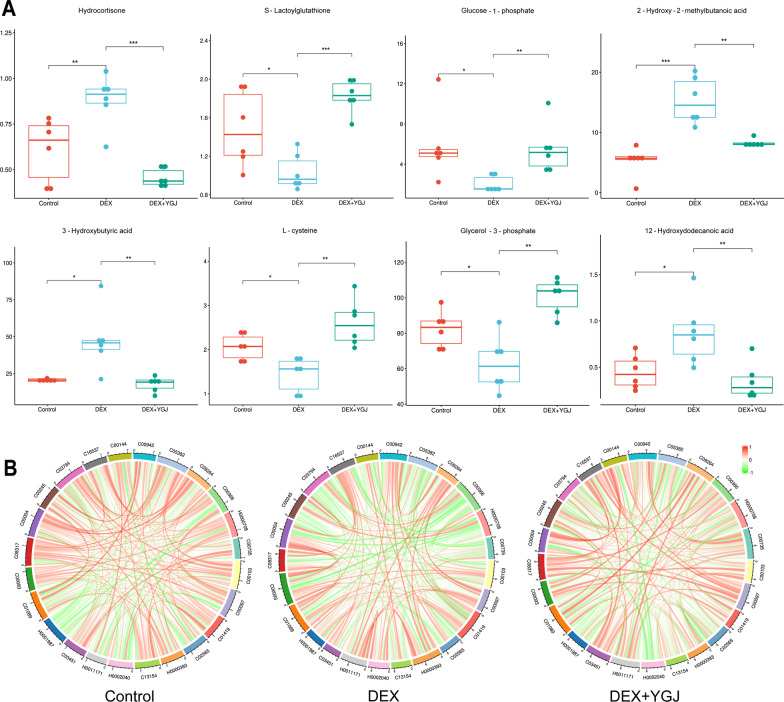
Box plots and correlation analysis of significant markers. **A** Box plots of the top 8 significant markers, *P < 0.05, **P < 0.01, ***P < 0.001, vs. DEX group; **B** correlation changes of 24 significant markers with themselves after DEX injection and YGJ intervention

Significant markers were mapped to the database for identification analysis and 24 of these metabolites had biological information (Table 1), suggesting the need to focus on the pathways in which these significant markers are located. Significant pullback occurred in hydrocortisone, s-lactoylglutathione, glucose-1-phosphate, 2-hydroxy-2-methylbutanoic acid, 3-hydroxybutyric acid, l-cysteine, glycerol- 3-phosphate, 12-hydroxydodecanoic acid, etc. (Fig. 5A). The Pearson correlation analysis was continued for the 24 significant markers (Fig. 5B), it was observed that the metabolite correlations changed between the groups with positive correlations becoming irrelevant or negative. After YGJ treatment, some of the correlation change caused by GIOP was reversed.

**Table 1:** 24 markers of liver metabolomics in YGJ-balanced GIOP mice

No	Metabolites	log_2_(FC)	*P*-value	VIP	KEGG	HMDB	Change
1	Hydrocortisone	− 0.95252	0.00005	2.69217	C00735	HMDB0014879	↓
2	S-Lactoylglutathione	0.81368	0.00011	2.35069	C03451	HMDB0001066	↑
3	Glucose-1-phosphate	1.45230	0.00110	2.38254	C00103	HMDB0001586	↑
4	2-Hydroxy-2-methylbutanoic acid	− 0.88685	0.00175	2.22642	–	HMDB0001987	↓
5	3-Hydroxybutyric acid	− 1.41490	0.00213	2.06207	C01089	HMDB0000357	↓
6	L-cysteine	0.85630	0.00255	2.31758	C00097	HMDB0000574	↑
7	Glycerol-3-phosphate	0.68772	0.00263	2.12763	C00093	HMDB0000126	↑
8	12-Hydroxydodecanoic acid	− 1.33550	0.00427	1.96774	C08317	HMDB0002059	↓
9	Reduced nicotinamide adenine dinucleotide	1.13700	0.00450	2.03607	C00004	HMDB0001487	↑
10	Taurine	− 0.63548	0.00514	1.93328	C00245	HMDB0000251	↓
11	Adenylosuccinic acid	1.53770	0.00550	1.91842	C03794	HMDB0000536	↑
12	Cys-Gly	0.67538	0.00700	2.15769	C01419	HMDB0000078	↑
13	Pentadecanoic acid	− 0.52265	0.00758	1.86792	C16537	HMDB0000826	↓
14	GMP	1.02350	0.01049	1.83988	C00144	–	↑
15	N-Methylhydantoin	− 0.81675	0.01147	2.06094	C02565	HMDB0003646	↓
16	cGMP	− 2.13940	0.01447	1.81362	C00942	HMDB0001314	↓
17	D-Sedoheptulose-7-phosphate	− 0.67165	0.01884	1.78553	C05382	HMDB0001068	↓
18	Trans-2-Butene-1,4-dicarboxylic acid	0.92075	0.01888	1.94288	–	HMDB0000393	↑
19	Dehydrocholic acid	− 0.42477	0.02335	1.97122	C13154	–	↓
20	Esculin	0.62726	0.02685	1.63272	C09264	HMDB0030820	↑
21	Uric acid	0.42277	0.02799	1.62877	C00366	HMDB0000289	↑
22	4-Methoxycinnamic acid	0.56239	0.02934	1.85603	–	HMDB0002040	↑
23	Glycoursodeoxycholic acid	− 0.61274	0.03382	1.58249	–	HMDB0000708	↓
24	Gamma-Glu-Leu	0.73205	0.03719	1.76303	–	HMDB0011171	↑

### Analysis of the metabolic pathways

**Fig. 6:**
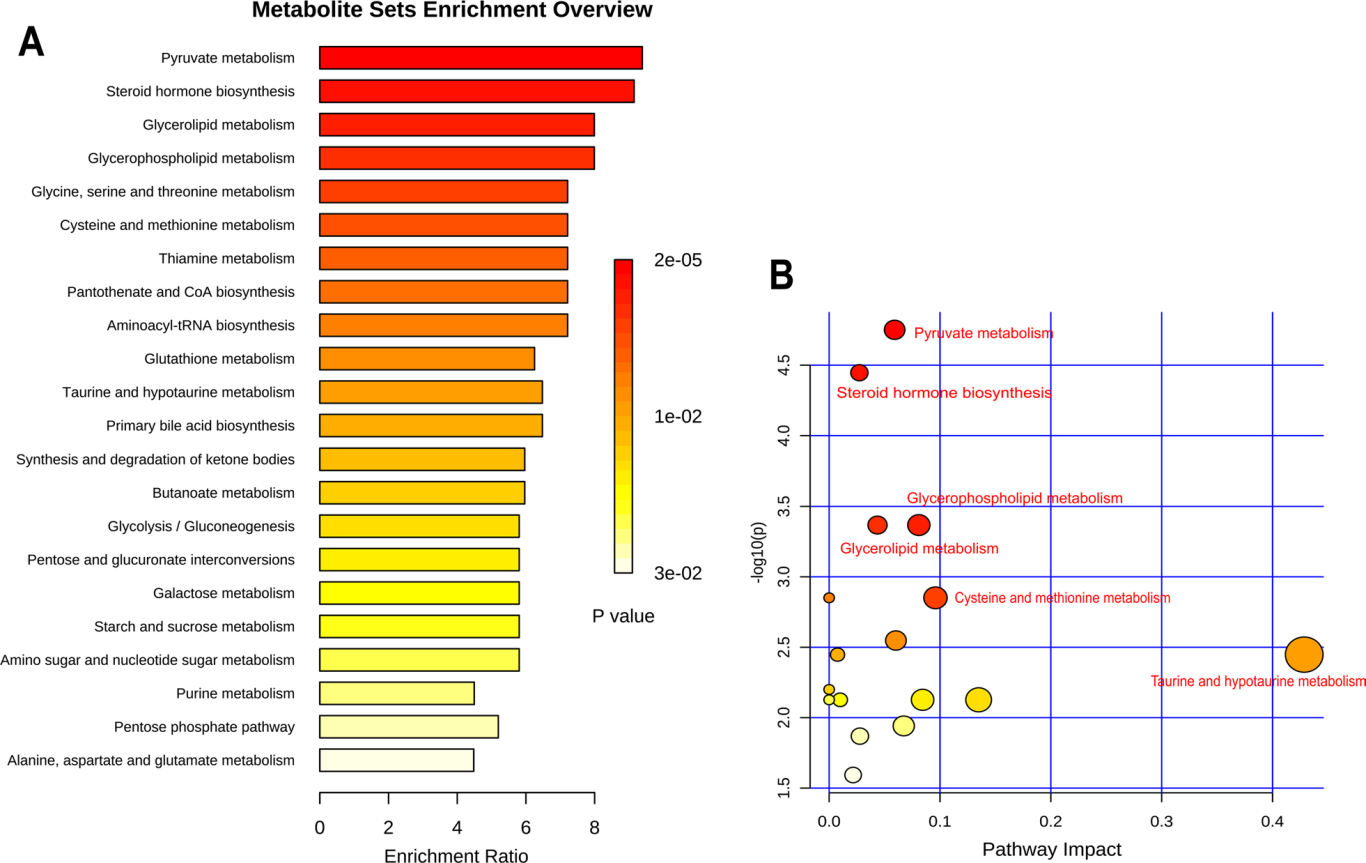
Metabolic pathways involved in YGJ-regulated metabolic disorders in the liver of GIOP mice. **A** Pathway enrichment analysis of 24 significant markers; **B** analysis of the effects of metabolic pathways on 24 significant markers; **C** partial taurine and hypotaurine metabolic pathways

Referring to the KEGG database, 24 markers with significant differences were mapped into the database for metabolic pathway analysis. Metabolic pathways enriched with significant markers are shown in Fig. 6A, which involve pyruvate metabolism, steroid hormone biosynthesis and glycerolipid metabolism, glycine, serine and threonine metabolism, taurine and hypotaurine metabolism, and other pathways. To further explore the mechanism of YGJ regulation of metabolic disorders in GIOP mice, the metabolic pathways were analyzed using Metabo Analyst. The results showed that the effect of YGJ on taurine and hypotaurine metabolism was more significant, − log_10_ (*P*) > 2.0 and Impact > 0.4 (Fig. 6B).

**Fig. 7:**
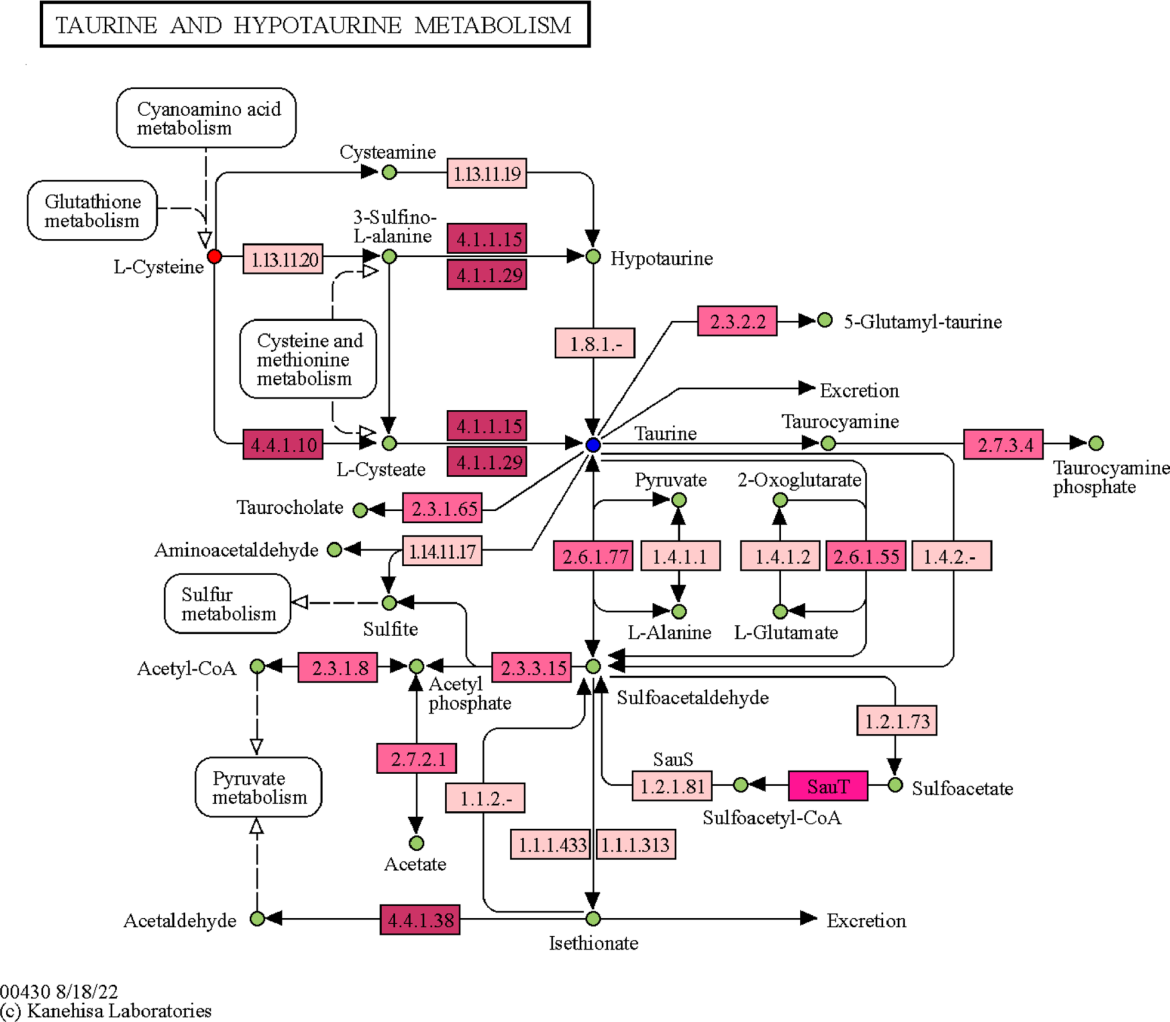
Taurine and hypotaurine metabolism pathway

Based on this, the metabolic pathways that YGJ may be involved in were further mapped with reference to the KEGG taurine and hypotaurine metabolism pathway (Fig. 7). In the map, it can be seen that YGJ affects the taurine content downstream by upregulating L-cysteine level upstream. Therefore, it may involve interference ①L-cysteate; ②cysteamine → hypotaurine; ③3-sulfoyl-L-alanine → hypotaurine; ④3-sulfoyl-L-alanine → L-cysteate and their related enzymes affect the metabolic pathways of taurine and hypotaurine, so as to play the role of balancing bone metabolism.

## Discussion

The aim of this study was to assess the role of YGJ on the progression of DEX-induced bone loss in the GIOP mouse model, which was prepared mainly by long-term administration of corticosteroids in mice in excess of physiological doses [9]. Hormones can inhibit osteoblast activity and promote bone resorption, while causing a decrease in bone formation as well as changes in calcium, phosphorus, and vitamin D, which leads to bone loss and induces osteoporosis [10]. In this study, after 8 weeks of intramuscular injection of DEX at a dose of 5 mg/kg three times a week [11], mice in the DEX group showed obvious bone loss manifestations such as decreased bone density and decreased number of bone trabecular with increased gaps compared with mice in the Control group. The subsequent pathological section results also occurred the same results, presenting a widespread decrease in osteocytes and bone trabeculae fracture. However, the YGJ intervention was able to reverse bone density loss and improve bone microarchitecture, and also showed denser bone trabecular morphology under light microscopy. That suggesting YGJ administration can effectively alleviate bone loss and rescue bone microarchitecture deterioration.

The specific pathogenesis of GIOP is still unclear; it was believed that the bone loss in GIOP was mainly due to decreased bone formation function accompanied by enhanced bone resorption in previous studies [12]. To further demonstrate that YGJ has an effective about anti-GIOP, bone conversion-related marker assays were experimentally performed. ALP is produced by osteoblasts during bone formation, and it hydrolyzes phosphate to provide the necessary phosphate for hydroxyapatite deposition and hydrolyzes pyrophosphate to relieve its inhibitory effect on bone salt formation, the level of which is a reflection of bone formation function and a tool to monitor the effectiveness of treatment in patients with osteoporosis [13, 14]. In this study, the YGJ group improved the serum ALP levels that were reduced by DEX after treatment. In addition to this, OCN is more sensitive to the effects of bone formation function than ALP, a non-collagenic protein secreted by osteoblasts, which are mostly embedded in the bone matrix. It contains three γ-carboxyglutamate residues, which have high affinity to the bone hydroxyapatite matrix [15], and the measurement of OCN reflects both osteoblast activity and the degree of bone conversion, and OCN has been found to be a useful biomarker for GIOP [16, 17]. Our results showed that serum OCN levels in the YGJ group were significantly higher than those in the DEX group, and the difference was statistically significant, suggesting that YGJ improves bone matrix mineralization levels and enhances bone formation. For markers of bone resorption we focused on CTX-I, which is formed by the degradation of the C-terminus of type I collagen by cathepsin K, which has now been recommended by the International Osteoporosis Foundation as a reference index reflecting bone resorption [18]. After 8 weeks of DEX injection, the serum CTX-I levels increased more significantly than in the Control group. After ALN intervention, CTX-I levels were lower but not significantly lower than in the DEX group. The baseline reduction in CTX-I after YGJ intervention with did not change observably. Previous studies have shown that CTX-I fluctuates by diurnal time changes and diet, peaking at 5:00 and dropping to a minimum at 14:00 [19]. Although we had fasted the mice prior to blood collection, we regrettably missed the optimal time for serum collection the following morning, resulting in no evidence for the anti-bone resorption effect of YGJ on GIOP mice in this study.

To characterize the underlying mechanisms of the preventive effect of YGJ on GIOP, a metabolomics analysis of mouse liver based on LC–MS analysis was applied to the mouse liver and 24 potential biomarkers were identified. Cortisol, a type of glucocorticoid, is known to induce osteoporosis in long-term administration [20]. Supraphysiological doses of glucocorticoids in vivo affect bone turnover by inhibiting osteoblast activity, inhibiting calcium absorption, stimulating bone resorption and renal excretion, and reducing skeletal angiogenesis [21–23], leading to a rapid decrease in bone mineral density, altering bone structure, and increasing fracture risk. In liver metabolites, higher levels of cortisol were found in the DEX group, while YGJ reversed the abnormal rise after 8 weeks of treatment, suggesting that YGJ can balance cortisol levels and further maintain bone homeostasis.

In addition to this, among the metabolites altered by dexamethasone intervention, 3-hydroxybutyric acid, taurine, esculin, uric acid, and other related metabolites have been shown in previous studies to be strongly associated with bone metabolism. 3-hydroxybutyric acid and its derivatives down-regulate the nuclear factor of T cell cytoplasmic 1 (NFATc1), a transcription factor that activates predifferentiation of osteoclasts. When NFATc1 activation and downstream function are inhibited, is able to reduce osteoclast predifferentiation and thus prevent bone resorption [24]. These that attenuate the effects of bone resorption also include esculin, which attenuates MAPK activation and NF-κB activity upon RANKL induction, leading to a reduction in Nfatc1 mRNA expression to regulate bone metabolism by inhibiting osteoclastogenesis and related transduction signals to regulate bone metabolism [25]. One of the risk factors for bone loss is oxidative stress, which mainly manifests itself by inhibiting osteoblast proliferation and differentiation and affecting bone matrix mineralization, inducing osteoblast apoptosis while enhancing osteoclast activity, resulting in more bone resorption [26]. Taurine, as an endogenous antioxidant, has anti-inflammatory, antioxidant, calcium ion regulation, and hypoglycemic effects [27]. Some researchers have focused on taurine for its role in the treatment of osteoporosis, showing that reduced taurine synthesis leads to dysregulation of calcium and vitamin D, which are essential elements for bone growth and resorption [28]. There is also evidence that taurine promotes osteoclastogenesis and increase ALP activity and calcium deposition [29]. In addition, the regulatory effects of taurine on osteoclasts and osteoblasts have also been explained [30]. Uric acid is a more powerful endogenous antioxidant, but the role of uric acid in osteoporosis remains controversial. Studies have reported that uric acid is able to reduce the level of reactive oxygen species in osteoclast precursors and promote the proliferation and differentiation of human bone mesenchymal stem cells into osteoblasts [31]. However, some data do not support this assertion; they suggest that uric acid increases the risk of fracture and that vitamin D deficiency and consequent secondary hyperparathyroidism due to high uric acid can further increase bone resorption and aggravate bone loss [32]. All of the above metabolites showed varying levels of elevation or reduction after DEX intervention. Our findings suggest that YGJ can balance the disorder of metabolite levels that are closely related to bone metabolism, and it is reasonable to speculate that the mechanism YGJ anti-GIOP may be related to the pathways in which these metabolites are located.

Further pathway analysis revealed that the taurine and hypotaurine metabolic pathways are of greater interest. The l-cysteine in this pathway was also back-regulated by YGJ in GIOP mice. Studies have also confirmed the effectiveness of it involved in bone marrow mesenchymal stem cell transplantation for osteoporosis [33], and in vitro experiments have shown that the pathways disturbed in osteoclasts treated with bisphosphonates also include the taurine and hypotaurine metabolic pathways [34], but current studies are limited. This study helps to validate the molecular mechanism of action of YGJ to regulate bone metabolic homeostasis, effectively leveraging the advantages of TCM, and further explore the long-term beneficial treatment of glucocorticoid osteoporosis.

## Conclusion

Yi-Guan-Jian decoction can increase bone density and improve bone microstructure by regulating the levels of alkaline phosphatase and osteocalcin and reverse bone loss in GIOP mouse model. The underlying metabolic mechanism may be related to taurine and hypotaurine metabolic pathway (Additional file 1).

## Supplementary Information

**Additional file 1: Fig. 1S**. YGJ affected the GIOP mice weight; **Table 1S**. Quantitative data of Micro-CT; **Table 2S**. Average positive optical density value; **Table 3S**. Effects of YGJ on ALP/OCN/CTX-I data in GIOP mice; **Table 4S**. YGJ reversed 52 differential metabolite data of GIOP.

## Data Availability

The datasets supporting the conclusions of this article are included within the article.

## Abbreviations

YGJ: Yi-Guan-Jian decoction
GIOP: Glucocorticoid-induced osteoporosis
DEX: Dexamethasone
ALN: Alendronate
TCM: Traditional Chinese Medicine
ROI: Region of interest
BMD: Bone mineral density
BMC: Bone mineral content
Tb. Sp: Trabecular separation
Tb. N: Trabecular number
Conn. D: Trabecular connection density
SMI: Structural pattern index
BV/TV: Bone volume/tissue volume
BS/TV: Bone superficial area/tissue volume
ALP: Alkaline phosphatase
OCN: Osteocalcin
CTX-I: C-terminal telopeptide of type-I collagen
QC: Quality control
PCA: Principal Component Analysis
PLS-DA: Partial Least Squares-Discrimination Analysis
OPLS-DA: Orthogonal Partial Least Squares-Discriminant Analysis
VIP: Variable Importance for Projection
NFATc1: Nuclear factor of T cell cytoplasmic 1
