# Supplementary material for: Metabolomics analysis of the potential mechanism of Yi-Guan-Jian decoction to reverse bone loss in glucocorticoid-induced osteoporosis
Source: J Orthop Surg Res. 2023 Jun 5;18:409. doi: 10.1186/s13018-023-03778-6 (PMC10242983; doi:10.1186/s13018-023-03778-6)
Supplement: Supplementary file 1 — Additional file 1: Fig. 1S. YGJ affected the GIOP mice weight; Table 1S. Quantitative data of Micro-CT; Table 2S. Average positive optical density value; Table 3S. Effects of YGJ on ALP/OCN/CTX-I data in GIOP mice; Table 4S. YGJ reversed 52 differential metabolite data of GIOP. [file 13018_2023_3778_MOESM1_ESM.docx]

**TITLE PAGE**

**Title:** Metabolomics analysis of the potential mechanism of Yi-Guan-Jian decoction to reverse bone loss in glucocorticoid-induced osteoporosis

**Authors:** Meng-Xing Yin ^1†^,De-Zhi Zhou ^1†^, Fu Jia ^2^ ^#^, Xiao-San Su ^1^, Xiu-Fang Li ^3^, Rui-Fen Sun ^1^, Jun-Min Li ^2^ ^#^

**Affiliation:** ^1^ Yunnan University of Chinese Medicine, Kunming, China; ^2^ Department of Orthopedics, Yunnan Hospital, Kunming Medical University, Kunming, China; ^3^ West Yunnan University of Applied Sciences, Dali, China

**Correspondence**^#^**:** Fu Jia. Kunming Yan 'an Hospital, department of orthopedics,245 Renmin Dong Lu, Kunming, China. Email: 524821887@qq.com; Jun-Min Li. Kunming Yan 'an Hospital, department of orthopedics,245 Renmin Dong Lu, Kunming, China. Email: lijunmin62@sina.cn.

**Running title:** Metabolomics analysis, YGJ treating GIOP

**Word Count:**4940

**Number of figures, videos and tables:**7

**Author Contributions:** (I) Conception and design: MX Yin, DZ Zhou, F Jia; (II) Administrative support: JM Li; (III) Provision of study materials or patients: XS Su, RF Sun; (IV) Collection and assembly of data: MX Yin, DZ Zhou; (V) Data analysis and interpretation: MX Yin, DZ Zhou, XF Li; (VI) Manuscript writing: All authors; (VII) Final approval of manuscript: All authors.

^†^ These authors contributed equally to this work.

**Contents**

**Fig. 1S.** YGJ affected the GIOP mice weight.

**Tab. 1S.** Quantitative data of Micro-CT.

**Tab. 2S.** Average positive optical density value.

**Tab. 3S.** Effects of YGJ on ALP/OCN/CTX-Ⅰdata in GIOP mice.

**Tab. 4S.** YGJ reversed 52 differential metabolite data of GIOP.

**Fig. 1S.** YGJ affected the GIOP mice weight. (*n*=15, *x ± s*)


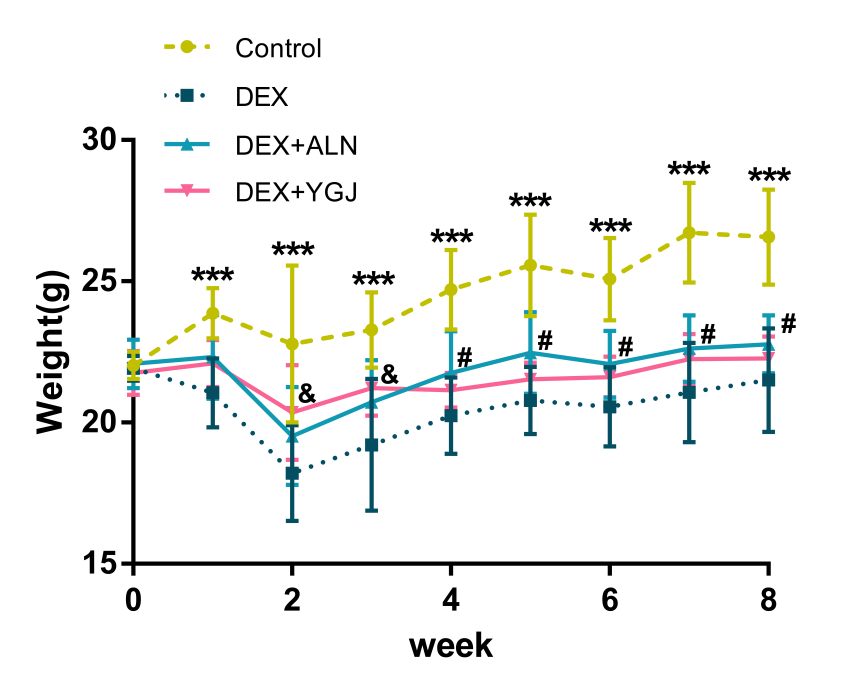


**P*＜0.05，** *P*＜0.01 ，*** *P*＜0.001，Control group *vs.* DEX group；

# *P*＜0.05，## *P*＜0.01 ，### *P*＜0.001，DEX+ALN group *vs.* DEX group；

& *P*＜0.05，&& *P*＜0.01 ，&&& *P*＜0.001，DEX+YGJ group *vs.* DEX group

**Tab. 1S.** Quantitative data of Micro-CT.

| **Quantitative data of Micro-CT** | | | | | | | | | | | | |
| --- | --- | --- | --- | --- | --- | --- | --- | --- | --- | --- | --- | --- |
|  | **Control** | | | **DEX** | | | **DEX+ALN** | | | **DEX+YGJ** | | |
|  | **C1** | **C2** | **C3** | **D1** | **D2** | **D3** | **A1** | **A2** | **A3** | **Y1** | **Y2** | **Y3** |
| **Tb.BV/TV** | 0.161044 | 0.144609 | 0.123825 | 0.069874 | 0.116272 | 0.05789 | 0.166144 | 0.154712 | 0.157134 | 0.152744 | 0.127465 | 0.126191 |
| **Tb.BS/TV(mm^-1)** | 9.29201 | 10.43651 | 8.76629 | 5.11345 | 7.68612 | 5.09373 | 10.4844 | 11.2527 | 11.0127 | 8.89315 | 8.03679 | 9.95095 |
| **Tb.Sp(mm)** | 0.197511 | 0.203735 | 0.199819 | 0.281542 | 0.221393 | 0.239363 | 0.184497 | 0.174506 | 0.182356 | 0.226721 | 0.205058 | 0.211363 |
| **Tb.N(mm^-1)** | 4.09312 | 4.05546 | 4.02911 | 3.01204 | 3.66312 | 3.57917 | 4.1934 | 4.53472 | 4.35645 | 3.71043 | 4.00029 | 3.88842 |
| **SMI** | 1.77312 | 1.74307 | 1.58749 | 1.75969 | 1.52772 | 1.81724 | 1.44536 | 1.28592 | 1.24565 | 1.4898 | 1.46023 | 1.70671 |
| **Conn.D(mm^-3)** | 167.0102 | 114.049 | 132.8059 | 96.4719 | 97.1019 | 67.9112 | 161.724 | 203.845 | 194.097 | 131.91 | 124.449 | 123.7834 |
| **Tb.BMD(g/cm^3)** | 1.3197 | 1.30052 | 1.30917 | 1.25718 | 1.25485 | 1.23015 | 1.29501 | 1.29024 | 1.3463 | 1.30215 | 1.31114 | 1.29517 |
| **Tb.BMC(mg)** | 1.8016 | 1.71471 | 1.75027 | 1.44304 | 1.52297 | 1.26799 | 1.66408 | 1.81024 | 1.73406 | 1.77474 | 1.70136 | 1.65242 |

**Tab. 2S.** Average positive optical density value.

| **Average Positive Optical Density Value ( lux )** | | | |
| --- | --- | --- | --- |
|  | **OD1** | **OD2** | **OD3** |
| **Control** | 1.381775 | 1.425721 | 1.371083 |
| **DEX** | 1.264797 | 1.230148 | 1.210425 |
| **DEX+ALN** | 1.343211 | 1.388793 | 1.393159 |
| **DEX+YGJ** | 1.398212 | 1.365372 | 1.29987 |

**Tab. 3S.** Effects of YGJ on ALP/OCN/CTX-Ⅰdata in GIOP mice.

| **Effects of YGJ on ALP\OCN\CTX-Ⅰdata in GIOP mice** | | | | | | | | | | | | |
| --- | --- | --- | --- | --- | --- | --- | --- | --- | --- | --- | --- | --- |
|  | **Control** | | | **DEX** | | | **DEX+ALN** | | | **DEX+YGJ** | | |
|  | **C1** | **C2** | **C3** | **D1** | **D2** | **D3** | **A1** | **A2** | **A3** | **Y1** | **Y2** | **Y3** |
| **ALP (U/L)** | 10.06085 | 10.17422 | 8.611204 | 7.141547 | 5.680227 | 5.317606 | 10.17089 | 8.892131 | 9.417306 | 7.663388 | 7.663388 | 6.747249 |
| **OCN (ng/mL)** | 78.64068 | 72.71863 | 67.12928 | 55.00951 | 49.04943 | 49.36312 | 89.85741 | 78.0038 | 79.10646 | 66.85361 | 61.73004 | 56.86312 |
| **CTX-1 (ng/mL)** | 0.588448 | 0.222924 | 0.467509 | 0.613718 | 0.855596 | 1.290614 | 0.944946 | 0.648014 | 0.927798 | 1.009025 | 0.8213 | 1.001805 |

**Tab. 4S.** YGJ reversed 52 differential metabolite data of GIOP.

| **YGJ reversed 52 differential metabolite data of GIOP** | | | | | | | | | | | | | | | | | | | |
| --- | --- | --- | --- | --- | --- | --- | --- | --- | --- | --- | --- | --- | --- | --- | --- | --- | --- | --- | --- |
| **Group** | **Control** | | | | | | **DEX** | | | | | | **DEX+YGJ** | | | | | |  |
| **Sample** | **C-1** | **C-2** | **C-3** | **C-4** | **C-5** | **C-6** | **D-1** | **D-2** | **D-3** | **D-4** | **D-5** | **D-6** | **Y-1** | **Y-2** | **Y-3** | **Y-4** | **Y-5** | **Y-6** |  |
| **Hydrocortisone** | 0.387 | 0.706 | 0.782 | 0.617 | 0.752 | 0.405 | 0.938 | 0.889 | 0.856 | 1.039 | 0.943 | 0.625 | 0.521 | 0.513 | 0.410 | 0.433 | 0.441 | 0.415 |  |
| **Glucose-1-phosphate** | 5.154 | 12.438 | 4.661 | 2.211 | 5.554 | 5.049 | 1.499 | 1.575 | 3.021 | 3.027 | 1.467 | 1.503 | 5.489 | 5.758 | 10.078 | 3.428 | 4.851 | 3.479 |  |
| **PC (14:1e/2:0)** | 33.616 | 42.168 | 45.294 | 22.094 | 29.694 | 38.363 | 10.627 | 20.148 | 22.443 | 11.427 | 22.847 | 31.476 | 30.918 | 38.992 | 60.623 | 56.107 | 45.997 | 52.181 |  |
| **L-cysteine** | 2.054 | 2.089 | 2.426 | 1.730 | 1.736 | 2.350 | 1.790 | 1.548 | 1.578 | 1.802 | 0.943 | 0.960 | 2.777 | 2.865 | 3.436 | 2.180 | 2.041 | 2.310 |  |
| **LPC 18:3** | 49.328 | 39.360 | 77.641 | 34.166 | 36.778 | 31.523 | 16.043 | 24.831 | 28.597 | 19.047 | 20.471 | 32.877 | 28.432 | 32.754 | 70.155 | 63.930 | 49.272 | 43.058 |  |
| **PC (18:5e/2:0)** | 25.344 | 29.156 | 48.116 | 20.192 | 21.408 | 17.379 | 5.387 | 14.124 | 13.906 | 14.536 | 15.282 | 24.210 | 18.991 | 25.001 | 65.443 | 38.843 | 35.770 | 29.404 |  |
| **YGJ reversed 52 differential metabolite data of GIOP** | | | | | | | | | | | | | | | | | | |  |
| **Group** | **Control** | | | | | | **DEX** | | | | | | **DEX+YGJ** | | | | | |  |
| **Sample** | **C-1** | **C-2** | **C-3** | **C-4** | **C-5** | **C-6** | **D-1** | **D-2** | **D-3** | **D-4** | **D-5** | **D-6** | **Y-1** | **Y-2** | **Y-3** | **Y-4** | **Y-5** | **Y-6** |  |
| **Cys-Gly** | 2.937 | 3.955 | 4.578 | 3.463 | 4.013 | 5.520 | 4.459 | 2.222 | 2.411 | 2.610 | 2.100 | 2.091 | 4.556 | 4.334 | 4.985 | 2.889 | 4.193 | 4.423 |  |
| **PC (18:3e/2:0)** | 52.533 | 55.416 | 79.106 | 38.506 | 50.090 | 49.449 | 10.048 | 31.655 | 43.584 | 26.698 | 33.749 | 27.219 | 52.997 | 54.629 | 84.846 | 52.192 | 55.891 | 70.298 |  |
| **LPC 14:0** | 2.365 | 2.349 | 3.801 | 1.939 | 2.409 | 2.352 | 1.105 | 2.214 | 1.790 | 1.206 | 1.336 | 2.400 | 2.273 | 2.634 | 4.329 | 2.031 | 2.926 | 2.856 |  |
| **N-Methylhydantoin** | 0.279 | 0.204 | 0.377 | 0.212 | 0.332 | 0.248 | 0.827 | 0.409 | 0.366 | 0.364 | 0.400 | 0.320 | 0.335 | 0.276 | 0.192 | 0.187 | 0.272 | 0.262 |  |
| **Lysopc 20:4** | 0.873 | 1.438 | 1.703 | 1.042 | 1.245 | 1.103 | 0.384 | 0.712 | 0.942 | 0.751 | 0.561 | 0.590 | 0.832 | 1.039 | 1.277 | 0.796 | 0.934 | 1.441 |  |
| **Cortisol** | 6.830 | 3.800 | 4.105 | 4.512 | 4.057 | 3.161 | 6.961 | 17.518 | 9.903 | 8.258 | 13.101 | 2.945 | 2.888 | 0.944 | 0.652 | 6.015 | 4.441 | 3.273 |  |
| **Trans-2-Butene-1,4-dicarboxylic Acid** | 2.041 | 3.792 | 2.667 | 2.099 | 2.358 | 3.358 | 1.926 | 1.034 | 2.227 | 1.867 | 1.386 | 1.199 | 1.813 | 3.425 | 5.867 | 2.295 | 2.389 | 2.459 |  |
| **PC (18:4e/4:0)** | 6.421 | 7.608 | 7.956 | 5.269 | 7.149 | 6.598 | 1.270 | 4.992 | 4.694 | 3.188 | 2.661 | 3.249 | 5.157 | 5.036 | 11.798 | 5.477 | 5.417 | 5.817 |  |
| **Lysopc 16:2 (2N Isomer)** | 0.270 | 0.076 | 0.113 | 0.094 | 0.131 | 0.101 | 0.047 | 0.060 | 0.026 | 0.039 | 0.109 | 0.104 | 0.069 | 0.106 | 0.156 | 0.107 | 0.197 | 0.131 |  |
| **PC (18:4e/2:0)** | 753.93 | 744.92 | 950.07 | 584.63 | 651.81 | 675.57 | 217.47 | 481.04 | 550.50 | 466.97 | 423.72 | 412.00 | 550.59 | 646.74 | 862.38 | 554.96 | 533.54 | 679.64 |  |
| **Dehydrocholic acid** | 0.733 | 0.460 | 0.384 | 0.654 | 0.867 | 0.651 | 1.002 | 1.092 | 1.102 | 0.962 | 0.839 | 0.950 | 0.484 | 0.847 | 0.704 | 0.965 | 0.640 | 0.790 |  |
| **LPC 17:2** | 0.448 | 0.379 | 0.391 | 0.328 | 0.281 | 0.336 | 0.151 | 0.221 | 0.250 | 0.167 | 0.303 | 0.350 | 0.286 | 0.235 | 0.695 | 0.615 | 0.573 | 0.355 |  |
| **LPC 16:2** | 0.451 | 0.368 | 0.607 | 0.398 | 0.414 | 0.513 | 0.249 | 0.333 | 0.266 | 0.220 | 0.240 | 0.376 | 0.287 | 0.380 | 0.786 | 0.453 | 0.662 | 0.349 |  |
| **Cysteinylglycine** | 17.250 | 17.676 | 18.250 | 13.978 | 12.871 | 15.510 | 18.849 | 10.129 | 11.230 | 9.610 | 11.482 | 9.087 | 17.479 | 21.527 | 15.652 | 10.615 | 18.593 | 18.726 |  |
| **LPE 16:1** | 16.485 | 28.606 | 31.028 | 16.229 | 18.094 | 27.302 | 3.713 | 8.628 | 16.043 | 9.866 | 4.567 | 10.473 | 16.250 | 22.374 | 22.421 | 9.322 | 10.657 | 27.227 |  |
| **YGJ reversed 52 differential metabolite data of GIOP** | | | | | | | | | | | | | | | | | | |  |
| **Group** | **Control** | | | | | | **DEX** | | | | | | **DEX+YGJ** | | | | | |  |
| **Sample** | **C-1** | **C-2** | **C-3** | **C-4** | **C-5** | **C-6** | **D-1** | **D-2** | **D-3** | **D-4** | **D-5** | **D-6** | **Y-1** | **Y-2** | **Y-3** | **Y-4** | **Y-5** | **Y-6** |  |
| **4-Methoxycinnamic acid** | 1.067 | 1.250 | 0.933 | 0.988 | 1.025 | 0.932 | 0.421 | 1.029 | 0.679 | 0.621 | 0.996 | 0.744 | 0.879 | 1.108 | 1.390 | 0.851 | 1.078 | 1.325 |  |
| **ACar 12:1** | 0.290 | 0.163 | 0.252 | 0.325 | 0.454 | 0.409 | 0.732 | 0.507 | 0.389 | 0.953 | 0.350 | 0.339 | 0.474 | 0.206 | 0.131 | 0.262 | 0.128 | 0.462 |  |
| **PC (14:1e/3:0)** | 18.970 | 15.296 | 17.662 | 12.178 | 12.808 | 14.862 | 2.549 | 6.472 | 10.479 | 5.404 | 8.348 | 7.872 | 9.150 | 12.069 | 19.672 | 8.223 | 11.389 | 10.682 |  |
| **Gamma-Glu-Leu** | 1.784 | 2.403 | 1.617 | 1.269 | 1.438 | 1.302 | 0.475 | 0.837 | 0.871 | 0.443 | 1.052 | 1.200 | 0.794 | 1.074 | 2.157 | 1.432 | 1.461 | 1.181 |  |
| **LPE 17:1** | 1.587 | 1.807 | 1.679 | 1.426 | 1.468 | 1.692 | 0.235 | 0.718 | 1.222 | 0.417 | 0.420 | 0.565 | 1.424 | 1.435 | 1.299 | 0.524 | 0.678 | 1.348 |  |
| **L-Tyrosinemethylester** | 1.072 | 1.229 | 0.967 | 0.970 | 1.045 | 0.938 | 0.476 | 0.999 | 0.423 | 0.683 | 0.977 | 0.756 | 0.872 | 1.003 | 1.356 | 0.769 | 0.982 | 1.284 |  |
| **ACar 19:1** | 0.155 | 0.214 | 0.402 | 0.093 | 0.256 | 0.118 | 0.063 | 0.044 | 0.096 | 0.075 | 0.077 | 0.185 | 0.076 | 0.100 | 0.426 | 0.419 | 0.388 | 0.118 |  |
| **N-{6-[(7-chloro-4-quinazolinyl)oxy]-3-pyridinyl}-4-methoxybenzamide** | 0.548 | 0.359 | 0.534 | 0.905 | 0.690 | 0.538 | 1.217 | 0.843 | 1.404 | 0.897 | 1.615 | 1.388 | 0.521 | 0.358 | 0.405 | 0.470 | 0.612 | 0.404 |  |
| **S-Lactoylglutathione** | 1.603 | 1.921 | 1.923 | 1.250 | 1.005 | 1.198 | 1.328 | 1.205 | 0.930 | 0.912 | 0.860 | 0.994 | 1.781 | 1.999 | 1.875 | 1.784 | 1.978 | 1.532 |  |
| **2-Hydroxy-2-methylbutanoic acid** | 6.018 | 7.892 | 0.678 | 5.500 | 5.515 | 5.891 | 16.447 | 10.857 | 12.592 | 12.464 | 19.091 | 20.201 | 7.976 | 7.997 | 8.089 | 9.481 | 8.304 | 7.718 |  |
| **[4-(1H-indol-4-yl)piperazino](2-thienyl)methanone** | 0.125 | 0.139 | 0.540 | 0.121 | 0.270 | 0.119 | 1.285 | 0.414 | 0.979 | 0.672 | 1.166 | 1.592 | 0.212 | 0.411 | 0.352 | 0.128 | 0.496 | 0.272 |  |
| **YGJ reversed 52 differential metabolite data of GIOP** | | | | | | | | | | | | | | | | | | |  |
| **Group** | **Control** | | | | | | **DEX** | | | | | | **DEX+YGJ** | | | | | |  |
| **Sample** | **C-1** | **C-2** | **C-3** | **C-4** | **C-5** | **C-6** | **D-1** | **D-2** | **D-3** | **D-4** | **D-5** | **D-6** | **Y-1** | **Y-2** | **Y-3** | **Y-4** | **Y-5** | **Y-6** |  |
| **3-Hydroxybutyric acid** | 19.350 | 19.746 | 21.417 | 21.383 | 21.879 | 19.767 | 84.400 | 44.282 | 47.427 | 21.238 | 47.380 | 40.543 | 20.878 | 9.959 | 19.910 | 23.738 | 14.006 | 18.492 |  |
| **Glycerol-3-phosphate** | 80.679 | 85.905 | 97.454 | 69.898 | 72.060 | 87.211 | 53.475 | 52.224 | 70.017 | 69.176 | 44.763 | 86.208 | 103.55 | 85.894 | 104.23 | 108.34 | 111.42 | 91.992 |  |
| **N-[3-(aminosulfonyl)phenyl]-2,3-dihydro-1,4-benzodioxine-2-carboxamide** | 15.023 | 29.642 | 22.799 | 10.864 | 16.268 | 14.444 | 8.611 | 7.913 | 14.199 | 9.699 | 8.028 | 11.299 | 14.002 | 21.573 | 26.436 | 15.397 | 18.235 | 12.762 |  |
| **12-Hydroxydodecanoic acid** | 0.294 | 0.248 | 0.590 | 0.494 | 0.350 | 0.707 | 0.980 | 0.808 | 1.464 | 0.890 | 0.586 | 0.495 | 0.701 | 0.335 | 0.223 | 0.177 | 0.219 | 0.415 |  |
| **Reduced nicotinamide adenine dinucleotide** | 8.610 | 11.447 | 10.550 | 9.705 | 10.368 | 8.453 | 5.179 | 7.077 | 10.721 | 3.041 | 4.294 | 8.418 | 12.796 | 14.819 | 14.309 | 13.516 | 19.638 | 10.099 |  |
| **Taurine** | 476.68 | 509.20 | 460.23 | 370.92 | 357.46 | 431.93 | 625.20 | 429.53 | 745.97 | 516.29 | 639.45 | 544.05 | 388.67 | 422.31 | 368.45 | 378.88 | 460.22 | 234.83 |  |
| **N1-(1,3-diphenyl-1H-pyrazol-5-yl)-2-chlorobenzamide** | 0.521 | 0.432 | 0.715 | 0.408 | 0.434 | 0.404 | 3.006 | 1.101 | 1.348 | 0.740 | 1.300 | 0.900 | 0.572 | 0.637 | 0.356 | 0.546 | 0.758 | 0.380 |  |
| **Adenylosuccinic acid** | 50.381 | 70.156 | 61.628 | 49.733 | 31.716 | 33.253 | 15.886 | 4.870 | 18.164 | 12.182 | 10.712 | 9.120 | 19.330 | 69.461 | 25.062 | 21.333 | 49.173 | 21.589 |  |
| **Pentadecanoic acid** | 0.294 | 0.305 | 0.348 | 0.342 | 0.287 | 0.411 | 0.467 | 0.726 | 0.517 | 0.539 | 0.641 | 0.459 | 0.527 | 0.346 | 0.289 | 0.383 | 0.397 | 0.389 |  |
| **D-(+)-Mannose** | 259.69 | 514.01 | 222.12 | 105.60 | 163.04 | 179.07 | 82.674 | 61.804 | 111.07 | 87.770 | 97.907 | 153.23 | 140.24 | 273.15 | 417.84 | 181.39 | 258.20 | 117.36 |  |
| **Fumaric acid** | 2.768 | 2.979 | 2.162 | 2.599 | 2.519 | 2.557 | 2.928 | 3.834 | 2.923 | 2.874 | 2.856 | 3.547 | 3.907 | 5.083 | 3.154 | 4.083 | 5.309 | 4.876 |  |
| **YGJ reversed 52 differential metabolite data of GIOP** | | | | | | | | | | | | | | | | | | |  |
| **Group** | **Control** | | | | | | **DEX** | | | | | | **DEX+YGJ** | | | | | |  |
| **Sample** | **C-1** | **C-2** | **C-3** | **C-4** | **C-5** | **C-6** | **D-1** | **D-2** | **D-3** | **D-4** | **D-5** | **D-6** | **Y-1** | **Y-2** | **Y-3** | **Y-4** | **Y-5** | **Y-6** |  |
| **Guanosine monophosphate (GMP)** | 6.424 | 8.499 | 6.453 | 7.894 | 3.695 | 4.223 | 1.991 | 0.858 | 3.501 | 1.698 | 1.313 | 2.092 | 3.141 | 5.266 | 3.354 | 3.542 | 5.591 | 2.390 |  |
| **cGMP** | 0.038 | 0.065 | 0.290 | 0.038 | 0.093 | 0.049 | 0.382 | 0.123 | 0.311 | 0.261 | 0.340 | 1.620 | 0.079 | 0.217 | 0.098 | 0.083 | 0.149 | 0.064 |  |
| **D-Sedoheptulose 7-phosphate** | 25.431 | 25.952 | 30.808 | 31.676 | 23.562 | 35.173 | 88.441 | 61.574 | 56.521 | 67.876 | 33.494 | 58.568 | 43.335 | 45.345 | 32.157 | 39.574 | 37.105 | 32.551 |  |
| **Esculin** | 0.328 | 0.376 | 0.318 | 0.317 | 0.186 | 0.213 | 0.222 | 0.167 | 0.121 | 0.136 | 0.089 | 0.148 | 0.344 | 0.259 | 0.174 | 0.171 | 0.180 | 0.236 |  |
| **H-Trp-NH2.HCl** | 0.108 | 0.123 | 0.075 | 0.093 | 0.073 | 0.096 | 0.475 | 0.160 | 0.186 | 0.163 | 0.268 | 0.393 | 0.115 | 0.220 | 0.110 | 0.158 | 0.087 | 0.030 |  |
| **Uric acid** | 56.233 | 58.422 | 42.024 | 51.216 | 37.639 | 50.635 | 35.777 | 45.817 | 31.228 | 33.756 | 46.251 | 44.022 | 66.462 | 54.567 | 49.071 | 49.964 | 61.144 | 36.290 |  |
| **Glycoursodeoxycholic acid** | 0.032 | 0.309 | 0.338 | 0.201 | 0.098 | 0.263 | 0.452 | 0.733 | 0.282 | 0.488 | 0.483 | 0.395 | 0.196 | 0.297 | 0.273 | 0.386 | 0.412 | 0.289 |  |
| **Thromoboxane B1** | 0.697 | 0.890 | 0.458 | 0.788 | 0.642 | 0.707 | 0.652 | 0.590 | 0.418 | 0.382 | 0.507 | 0.527 | 0.475 | 0.903 | 0.720 | 0.862 | 0.639 | 0.610 |  |
| **JWH 018 N-(5-hydroxypentyl)β-D-Glucuronide** | 0.514 | 0.695 | 0.715 | 0.617 | 0.462 | 0.602 | 1.086 | 0.760 | 0.806 | 0.848 | 0.975 | 1.237 | 0.834 | 0.812 | 0.686 | 0.505 | 0.891 | 0.683 |  |
